# Supplementary figures and images for: mRNA decapping is an evolutionarily conserved modulator of neuroendocrine signaling that controls development and ageing
Source: eLife. 2020 May 5;9:e53757. doi: 10.7554/eLife.53757 (PMC7200159; doi:10.7554/eLife.53757)

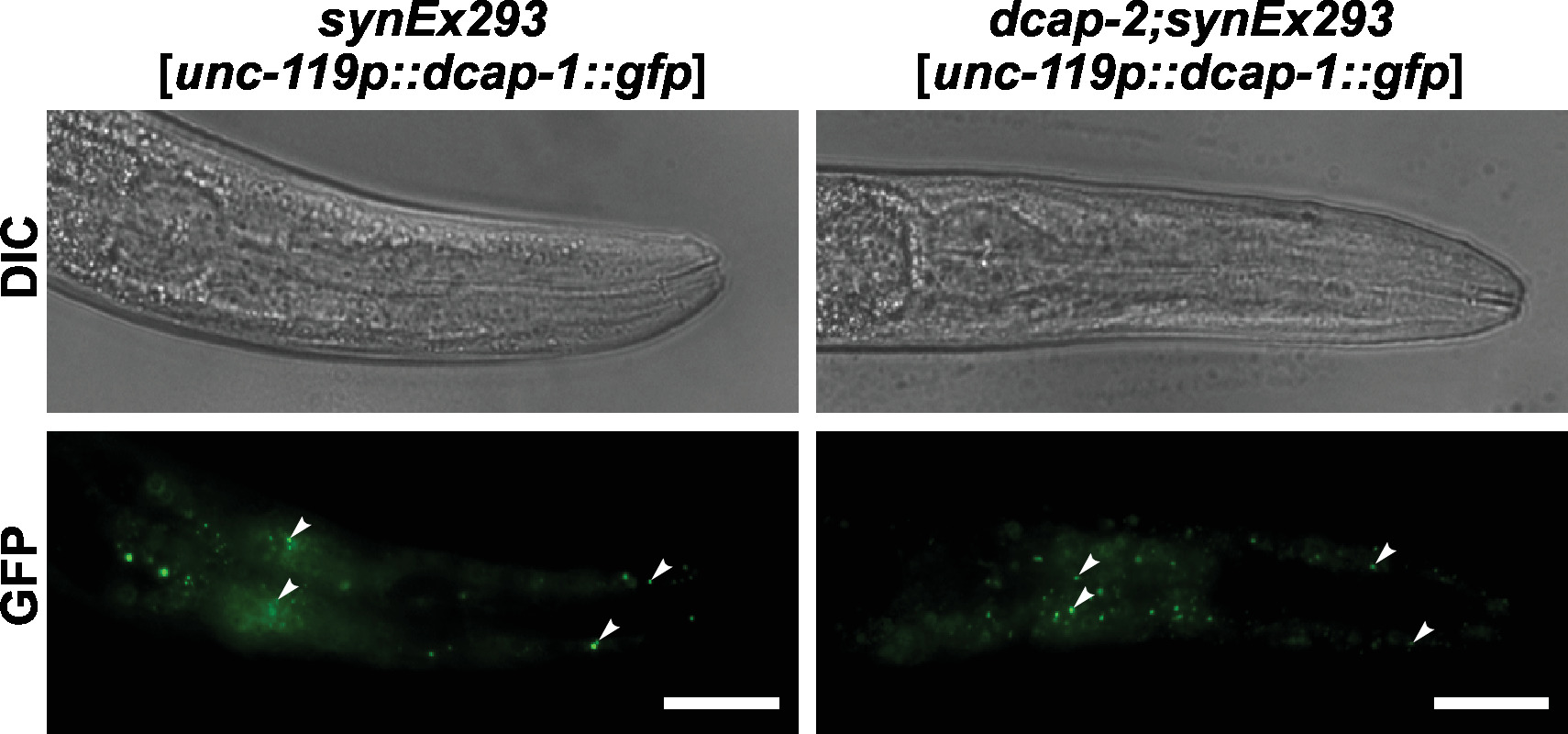

Supplement: Supplementary file 1. — Representative fluorescent images of wild type and dcap-2(ok3032) worms that express a dcap-1::gfp fusion under the control of the pan-neuronal unc-119 promoter. Arrowheads point to P-body like structures. Scale bar = 20 μm. [file elife-53757-supp1.jpg]

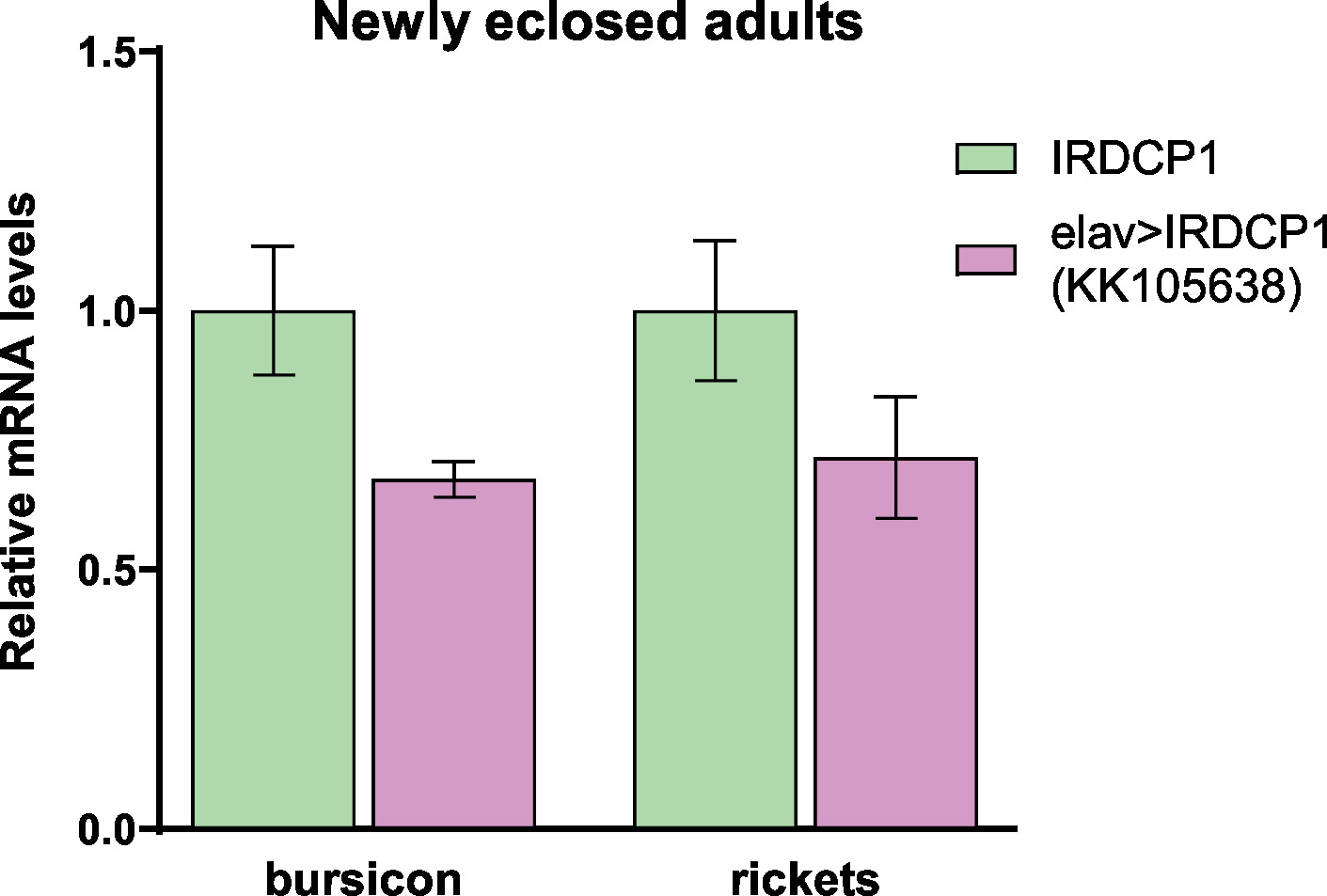

Supplement: Supplementary file 2. — Relative mRNA levels of bursicon and its receptor rickets in heads dissected form newly eclosed adults subjected to neuron-specific DCP1 knockdown at 29°C. [file elife-53757-supp2.jpg]
