# Supplementary material for: mRNA decapping is an evolutionarily conserved modulator of neuroendocrine signaling that controls development and ageing
Source: eLife. 2020 May 5;9:e53757. doi: 10.7554/eLife.53757 (PMC7200159; doi:10.7554/eLife.53757)
Supplement: Transparent reporting form [file elife-53757-transrepform.docx]

***eLife’s* transparent reporting form**

We encourage authors to provide detailed information *within their submission* to facilitate the interpretation and replication of experiments. Authors can upload supporting documentation to indicate the use of appropriate reporting guidelines for health-related research (see [EQUATOR Network](http://www.equator-network.org/%20)), life science research (see the [BioSharing Information Resource](https://biosharing.org/)), or the [ARRIVE guidelines](http://www.plosbiology.org/article/info:doi/10.1371/journal.pbio.1000412) for reporting work involving animal research. Where applicable, authors should refer to any relevant reporting standards documents in this form.

If you have any questions, please consult our Journal Policies and/or contact us: [editorial@elifesciences.org](mailto:editorial@elifesciences.org).

**Sample-size estimation**

- You should state whether an appropriate sample size was computed when the study was being designed
- You should state the statistical method of sample size computation and any required assumptions
- If no explicit power analysis was used, you should describe how you decided what sample (replicate) size (number) to use

Please outline where this information can be found within the submission (e.g., sections or figure legends), or explain why this information doesn’t apply to your submission:

For Lifespan analysis, we used size of >100 animals per strain, with only few experiments to have >50 animals. Sample sizes for survival assays are reported in Figure 1-Supplement file 1 and 2, Figure 2-Supplement files 1-3, Figure 3-Supplement file 7, Figure 5-Supplement 1.

For other experiments, sample sizes are shown in source files.

**Replicates**

- You should report how often each experiment was performed
- You should include a definition of biological versus technical replication
- The data obtained should be provided and sufficient information should be provided to indicate the number of independent biological and/or technical replicates
- If you encountered any outliers, you should describe how these were handled
- Criteria for exclusion/inclusion of data should be clearly stated
- High-throughput sequence data should be uploaded before submission, with a private link for reviewers provided (these are available from both GEO and ArrayExpress)

Please outline where this information can be found within the submission (e.g., sections or figure legends), or explain why this information doesn’t apply to your submission:

All experiments are replicated at least three times, otherwise noted in figure legends.

Biological replicates are different animals or groups of animals. Technical replicates are RNA samples from the same group of animals.

All replicates are biological replicates and only in qPCR experiments there were 2-3 technical replicates per biological replicate.

No animals, samples or lifespan replicates were excluded from the analyses.

Such information can be found in Figure legends, Figure supplement files or Materials and Methods section.

**Statistical reporting**

- Statistical analysis methods should be described and justified
- Raw data should be presented in figures whenever informative to do so (typically when N per group is less than 10)
- For each experiment, you should identify the statistical tests used, exact values of N, definitions of center, methods of multiple test correction, and dispersion and precision measures (e.g., mean, median, SD, SEM, confidence intervals; and, for the major substantive results, a measure of effect size (e.g., Pearson's r, Cohen's d)
- Report exact p-values wherever possible alongside the summary statistics and 95% confidence intervals. These should be reported for all key questions and not only when the p-value is less than 0.05.

Please outline where this information can be found within the submission (e.g., sections or figure legends), or explain why this information doesn’t apply to your submission:

For lifespan analyses, p values were calculated using Log-rank (Mantel-Cox) test.

For fluorescence quantification and qPCR, statistical significance was determined by two-tailed unpaired t-test unless otherwise indicated.

Error bars indicate mean ±SD

All p values are included in figure legends or lifespan analyses.

(For large datasets, or papers with a very large number of statistical tests, you may upload a single table file with tests, Ns, etc., with reference to sections in the manuscript.)

**Group allocation**

- Indicate how samples were allocated into experimental groups (in the case of clinical studies, please specify allocation to treatment method); if randomization was used, please also state if restricted randomization was applied
- Indicate if masking was used during group allocation, data collection and/or data analysis

Please outline where this information can be found within the submission (e.g., sections or figure legends), or explain why this information doesn’t apply to your submission:

For lifespan analyses, large groups of age-synchronized animals were prepared and ~100-120 were randomly selected per treatment group, divided into 3-4 separate plates.

For fluorescence quantification, large groups of age-synchronized animals were prepared and ~ 50-100 were randomly selected per treatment group, at the indicated ages.

For qPCR analyses, large groups of age-synchronized animals were prepared and ~ 150-300 were randomly selected per treatment group, at the indicated ages.

Masking was used in select experiments, during data collection or data analysis

**Additional data files (“source data”)**

- We encourage you to upload relevant additional data files, such as numerical data that are represented as a graph in a figure, or as a summary table
- Where provided, these should be in the most useful format, and they can be uploaded as “Source data” files linked to a main figure or table
- Include model definition files including the full list of parameters used
- Include code used for data analysis (e.g., R, MatLab)
- Avoid stating that data files are “available upon request”

Please indicate the figures or tables for which source data files have been provided:

List of source data:

Figure 3A, 3E, 3G and Figure 3-supplement 6: Numeric values of relative mRNA levels of insulin-like peptides in the indicated genetic backgrounds and ages.

(Figure 3-Source Data 1)

Figure 3B, 3C,3D, 3F and Figure 3-figure supplement 2A: Numeric values of relative fluorescence intensity of ins-7p::gfp and ges-1p::gfp reporters in the indicated genetic backgrounds.

(Figure 3-Source Data 2)

Figure 4A, 4B: Numerical values of DAF-16 positive nuclei plotted in panels A and B.

(Figure 4-Source Data 1)

Figure 4C, 4D, 4E and 4F: Numerical values of % survival plotted in panels C, D, E and F.

(Figure 4-Source Data 2)

Figure 5A and Figure 5-supplement 2: Numeric values of relative mRNA levels plotted in panel 5A and figure supplement 2.

(Figure 5-Source Data 1)
